# Association of hyperhomocysteinemia and chronic kidney disease in the general population: a systematic review and meta-analysis

**DOI:** 10.1186/s12882-023-03295-y

**Published:** 2023-08-23

**Authors:** Wei Chen, Jihua Feng, Pan Ji, Yani Liu, Huan Wan, Jianfeng Zhang

**Affiliations:** Guangxi Health Commission key Laboratory of Emergency and Critical Medicine, The Second Affiliated Hospital of Guangxi, Medical University, Nanning, China

**Keywords:** Homocysteine, Hyperhomocysteinemia, Chronic kidney disease

## Abstract

**Background:**

Increasing evidence shows that an elevated homocysteine(Hcy) level is associated with an increased risk of chronic kidney disease (CKD). This study systematically evaluated the correlation between homocysteine level and the incidence of CKD reported in cohort and cross-sectional studies.

**Methods:**

We searched electronic databases and reference lists for relevant articles. 4 cohort studies and 7 cross-sectional studies including 79,416 patients were analyzed in a meta-analysis. Hyperhomocysteinemia was defined as a Hcy level > 15 µmol/L, which was the criterium used in previous studies. Meta-analyses were conducted of literature searches from online databases such as PubMed, Embase, Cochrane and Scopus. Computed pooled adjusted odds ratios with corresponding 95% confidence intervals (95% CI) were used to estimate the risk of new-onset CKD according to Hcy levels in the general population.

**Results:**

People with high Hcy levels were more likely to suffer from CKD than people with normal Hcy levels (pooled OR, 2.09; 95% CI, 1.72–2.55). This positive relationship persisted across different study types such as cohort studies (summary OR, 2.2; 95% CI, 1.55–3.13) and cross-sectional studies (summary OR, 2.07; 95% CI, 1.63–2.63).

**Conclusions:**

People with hyperhomocysteinemia have a higher incidence of CKD, Hyperhomocysteinemia may also be an independent risk factor for CKD in the general population.

**Supplementary Information:**

The online version contains supplementary material available at 10.1186/s12882-023-03295-y.

## Background

Globally, 1.2 million people died from chronic kidney disease (CKD) in 2017. Global all-age mortality from CKD increased by 41 5% between 1990 and 2017; and kidney disease has had a major impact on global health, being both a direct cause of global morbidity and mortality and an important risk factor for cardiovascular disease. CKD is largely preventable and treatable and deserves more attention in global health policy decisions [1]. In Asia, it is estimated that as many as 434.3 million adults have CKD, of whom as many as 65.6 million have advanced CKD. The highest numbers of adults with CKD is in China (up to 159.8 million) and India (up to 140.2 million), which together account for 69.1% of adults with CKD in the region. Urgent collaborative action is therefore needed in Asia to prevent and manage CKD and its complications [2]. It is well established that the main site of homocysteine (Hcy) metabolism is in the kidneys with serum Hcy levels in patients with CKD being higher than those in patients with normal renal function [3]. A prospective study showed patients in the highest tertile of plasma Hcy levels had an increased incidence of CKD compared with those in the lower tertile [4]. Previous studies also showed that elevated plasma Hcy levels are associated with decreased GFR in patients with renal impairment, with GFR gradually decreasing with elevated Hcy levels. In addition, in the general population, elevated Hcy levels were reported to be associated significantly with decreased renal function, suggesting that this is a key risk factor for the development of CKD in the general population [5]. In a cohort study, tHcy concentration was found to be an independent determinant of eGFR changes. There was a graded association between tHcy quartiles and eGFR decline [6]. However, these results remain inconclusive due to the complex bidirectional interaction between changes in Hcy levels and renal function, while the coexistence of risk factors such as diabetes and hypertension [7, 8] complicates the definition of the role of Hcy. There are also limited data on the association between high Hcy levels and CKD risk in the general population. In addition, there is a lack of large randomized controlled studies demonstrating that Hcy levels have an impact on the incidence rate of CKD in the general population. Therefore, we performed a meta-analysis of studies to analyze the association of serum Hcy levels with the incidence of CKD in the general population. The aim of this study was to raise awareness of the importance of high Hcy levels in the development of CKD in the general population.

## Methods

### PRISMA statement

#### Eligibility criteria

The inclusion criteria were as follows: (1) report on the correlation between Hcy and new-onset CKD; (2) observational case-control studies, cross-sectional studies, or cohort design studies; (3) age ≥ 18 years, (4)report of the odds ratio (OR) or relative risk ratio (RR) in observational studies with 95% confidence intervals (95% CI) or sufficient information to calculate these figures. Exclusion criteria were: (1) studies published in languages other than English; (2) studies reporting patients with acute kidney injury or end-stage kidney disease or patients requiring dialysis. Studies without clear groupings or animal studies were excluded. Studies using other definitions of CKD, such as creatinine levels, were also excluded.

#### Information sources

The electronic databases used in this study were PubMed, Embase, Cochrane, and Scopus for published studies from establishment to June 2022. The electronic search was up to June 2022 with no restrictions on publication type.

#### Search strategy

Keywords used were the subject heading and a combination of free words, such as “hyperhomocysteinemia [Mesh] + free words”, “homocysteine” [Mesh] + freewords”, “renal insufficiency, chronic [Mesh] + free words” (see Appendix for details). The “related Items” function was used to expand the search.

#### Selection process

The studies were screened independently by two reviewers (Wei Chen and Ji-hua Feng), with disagreements resolved by discussion.

#### Data collection process

Two reviewers (Wei Chen and Ji-hua Feng) independently reviewed the studies with differences resolved through discussion. Data extraction included country of origin, study period, year of publication, inclusion criteria, definition of hyperhomocysteinemia or odds ratios for the prevalence of CKD in groups with different Hcy levels, adjusted for calculated variables in the analysis, and adjusted OR or RR estimates with corresponding 95% CI, patient characteristics, and conclusions. Hyperhomocysteinemia was defined as Hcy levels > 15 µmol/L, which varied in different studies [9, 10]. The primary outcome was the odds ratio (OR) for Hcy to predict the incidence of CKD. According to the National Kidney Foundation’s Kidney Disease Outcome and Quality Initiative (KDOQI) CKD guidelines [11], CKD was defined as an eGFR < 60 mL/min/1.73 m2 for more than 3 months, or the Renal injury markers, such as proteinuria, cystatin for longer than 3 months with or without a decrease in GFR. The incidence of CKD in prospective cohort studies was defined as individuals without CKD at baseline who experienced a decrease in GFR < 60 mL/min/1.73 m2 or hematuria and proteinuria for > 3 months during follow-up. In a cross-sectional study, the incidence of CKD was defined as individuals meeting the diagnostic criteria for CKD found in routine physical examinations by community residents and physical examination centers.

### Data items

If the study classified participants according to tertiles or quartiles of Hcy levels, the OR value was taken as the ratio of the highest quantile to the lowest quantile. If the study only listed the OR of men and women, the OR after the combination of men and women was taken as the OR of the study. The data were retrieved and merged to report the OR or RR of hyperhomocysteinemia and the risk of new-onset CKD.

### Study risk of bias assessment

The quality of the cohort studies and cross-sectional studies was assessed by two authors using the Newcastle-Ottawa Scale (NOS) and AHRQ, respectively. The NOS [12] assigned up to nine points for study population comparability, quality of selection, and outcomes, with study quality scores defined as poor (0–3), fair (4–6), or high (7–9). The AHRQ [13] consists of 11 items, with each item of the AHRQ answered as yes, no, or not reported, with only a “yes” answer scored as 1 and a “no” and “not reported” answers scored as 0. Scores of 4–6 were rated as medium quality and 8–11 points as high quality.

### Effect measures

Meta-analyses of the effect size results were performed using OR. All confidence intervals (CIs) were 95%. A p-value < 0.05 was considered statistically significant. Between-trial heterogeneity was assessed using the I^2 index and Q test p-value. If the p-value was < 0.05 and the I^2 index was > 50% this indicated the existence of heterogeneity between the studies [14].

### Synthesis methods

Possible sources of heterogeneity included various aspects, such as research methods, data types, sample size, study quality, and the characteristics of the participants including age, gender, location, blood pressure; HDL, high-density lipoprotein; LDL, low-density lipoprotein; FBG, fasting blood glucose; PBG, postprandial blood glucose; eGFR, estimated glomerular filtration rate;Hs-CRP, high-sensitivity C-reactive protein; SBP, systolic blood pressure; DBP, diastolic blood pressure; PP, pulse pressure. We explored the heterogeneity between studies using random-effects models, subgroup analyses, and meta-regression analyses. We used Stata version 12.0 for Windows8 for data analysis and graph formation.

### Reporting bias assessment

Publication bias was assessed by constructing funnel plots and Egger regression tests.

### Certainty assessment

Trimming and filling are used to evaluate the certainty (or credibility) in the body of evidence for an outcome.

## Results

**Fig. 1:**
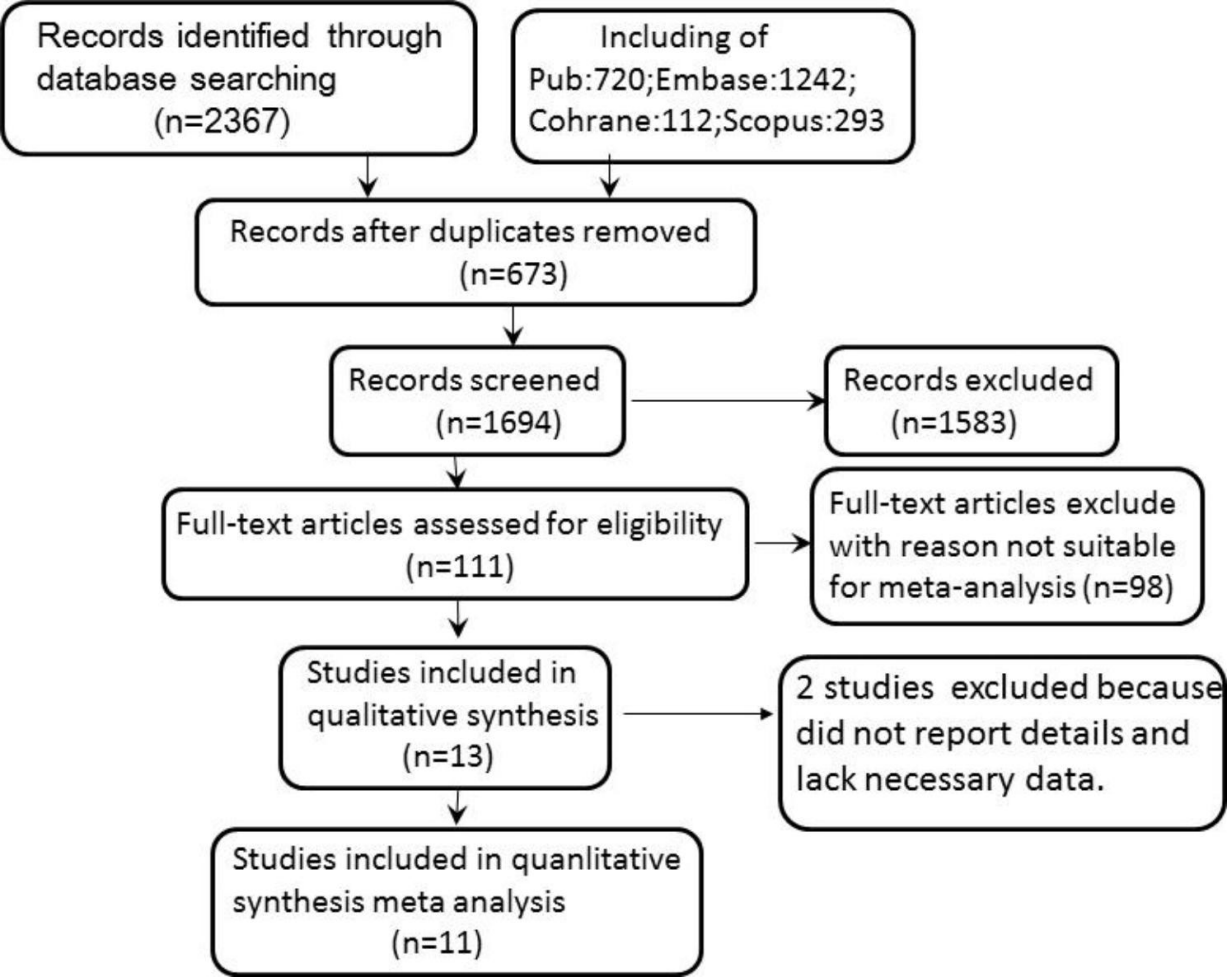
Flowchart of the study selection process

Figure 1 outlines the literature specific screening process. An electronic database search identified 2367 citations. After removing 673 duplicate literatures, 111 articles were selected for full-text review to understand their relevance to this study after reading the literature abstracts. In the full-text review stage, 27 articles did not reflect the relationship between Hcy and CKD, 24 articles were combined with other medical diseases, and 47 articles were reviews. Two studies were excluded from the main meta-analysis because they did not report details and the corresponding authors could not provide the necessary data. Finally, 11 studies were included in the systematic review. The consistency between researchers during the full-text review phase was excellent.

### Study characteristics

The characteristics of the 11 studies are shown in Table 1 [6, 15–24]. A total of 79,416 participants were included, with sample sizes ranging from 999 to 19,372. The 11 studies included 7 cross-sectional studies and 4 cohort studies, 9 of which were conducted in Asian populations, 1 from Israel, and 1 from Australia, with the subjects obtained from health check-ups or residents. According to the NOS literature quality evaluation Table 2 cohort studies showed relatively high quality (NOS > 6) and 2 cohort studies showed moderate quality (NOS = 6). According to the AHRQ literature quality evaluation Table 3 cross-sectional studies showed relatively high quality (AHRQ > 8).

The subjects studied were from the health check-up population or community residents.

**Table 1:** Characteristics of studies included in the meta-analysis

Author/ year	Country	Study type	Population (M/F)	Age (year)	HHcy (umol/L)	adOR (95%CI)	Adjustments	Quality evaluation NOS or AHRQ
Ninomiya 2004 [24]	Japan	Cohort study	1477 (596/881)	≥ 40	M > 10.6 F > 8.3	2.52 (1.21, 5.25)	Age, systolic blood pressure, habitual smoker, etc.	high
Shankar 2008 [23]	Australia	Cross sectional sutdy	2609 (1080/1529)	≥ 49	≥ 15	1.41 (1.06,1.88)	age, sex, smoking, alcohol, body mass index, etc.	high
Chuang 2013 [22]	China	Cross sectional study	19,372 (14,874/4498)	≥ 18	≥ 11.82	1.56 (1.13,2.15)	age, smoking, fasting glucose level,arterial pressure, etc.	high
Chao 2014 [21]	China	Cross sectional study	1581 (894/687)	≥ 18	≥ 12.24	6.73 (3.15,14.41)	Age, gender, alcohol diabetes smoking,, consumption, etc.	high
Levi 2014 [20]	Israel	Cross sectional study	3602 (2692/910)	≥ 20	≥ 15	3.2 (1.3, 7.6)	age, eGFR, mean BMI, mean HDL cholesterol, log-mean FA,, etc.	high
Chen 2015 [19]	China	Cross sectional study	999 (737/262)	≥ 18	≥ 10.1	4.85 (1.06,22.31)	Age,smoking, leptin, eGFR, arterial pressure, etc.,	high
Xie 2015 [18]	China	Cohort study	2387 (578/1809)	≥ 45	> 15	2.44 (1.26,4.72)	age, gender, SBP, diabetes, smoking, cholesterol, etc.	fair
Kong 2017 [17]	China	Cohort study	5917 (4410/1507)	≥ 40	≥ 15	1.6 (0.91,7.78)	age, sex, diabetes, BMI, uric acid, smoking, etc.	fair
Lai 2018 [16]	China	Cross sectional study	24,826 (19,076/5750)	≥ 18	> 11.81	2.12 (1.75,2.57)	gender, age, smoking, diabetes hypertension,etc.	high
Moon 2020 [15]	South Korea	Cross sectional study	15,220 (9059/6161)	≥ 19	≥ 15	1.98 (1.71,2.3)	smoking, drinking, physical activity, albumin level, etc.	high
Xiao 2021 [6]	China	Cohort study	1426 (607/819)	> 18	> 15	4.29 (1.42,12.99)	age, sex, diabetes mellitus, smoking, BMI, etc.	high

**Table 2:** Quality of the studies utilizing the Newcastle-Ottawa quality assessment scale (Cohort studies)

Reference (Year)	Selection				Comparability	Outcome			
	Representativeness of exposed cohort	Selection of the non-exposed cohort	Ascertainment of exposure	Demonstration outcome was not present at start of study	Comparability of cohorts on the basis of the design or analysis	Assessment of outcome	Follow up long enough	Adequacy of follow up of cohorts	Total score
Kong (2017)	$\bigstar$	$\bigstar$	$\bigstar$	$\bigstar$	$\bigstar$	$\bigstar$			6
Xiao (2021)	$\bigstar$	$\bigstar$	$\bigstar$	$\bigstar$	$\bigstar \bigstar$	$\bigstar$		$\bigstar$	8

**Table 3:** Quality of the studies utilizing the AHRQ quality assessment scale (cross-sectional studies)

Reference (Year)	①	②	③	④	⑤	⑥	⑦	⑧	⑨	⑩	⑪	Total score
Chao 2014	1	1	1	0	1	1	1	1	0	1	1	9
Levi 2014	1	1	1	0	1	1	0	1	1	1	1	9
Chen 2015	1	1	1	0	1	1	1	0	1	1	1	9
Lai 2018	1	1	1	0	1	1	1	1	1	0	1	9
Moon 2020	1	1	1	0	1	1	1	1	1	1	1	10

AHRQ: ① Whether the source of the data has been clarified (investigation, literature review);② Include and exclude criteria for both exposed and non exposed groups (case and control) or refer to previous publications;③ Whether a time period for identifying patients has been provided;④ If it is not a population source, whether the research object is continuous;⑤ Whether the subjective factors of the evaluator conceal other aspects of the research object;⑥ Describe any evaluation conducted to ensure quality (such as testing/retesting of primary outcome indicators);⑦ To explain the reasons for excluding any patients from the analysis;⑧ Describe how to evaluate (or control measures for confounding factors;⑨ Explained how to handle lost data in the analysis if possible;⑩ To summarize the response rate of patients and the completeness of data collection;⑪ To identify the percentage of expected patients with incomplete data or follow-up results if follow-up is available

**Fig. 2:**
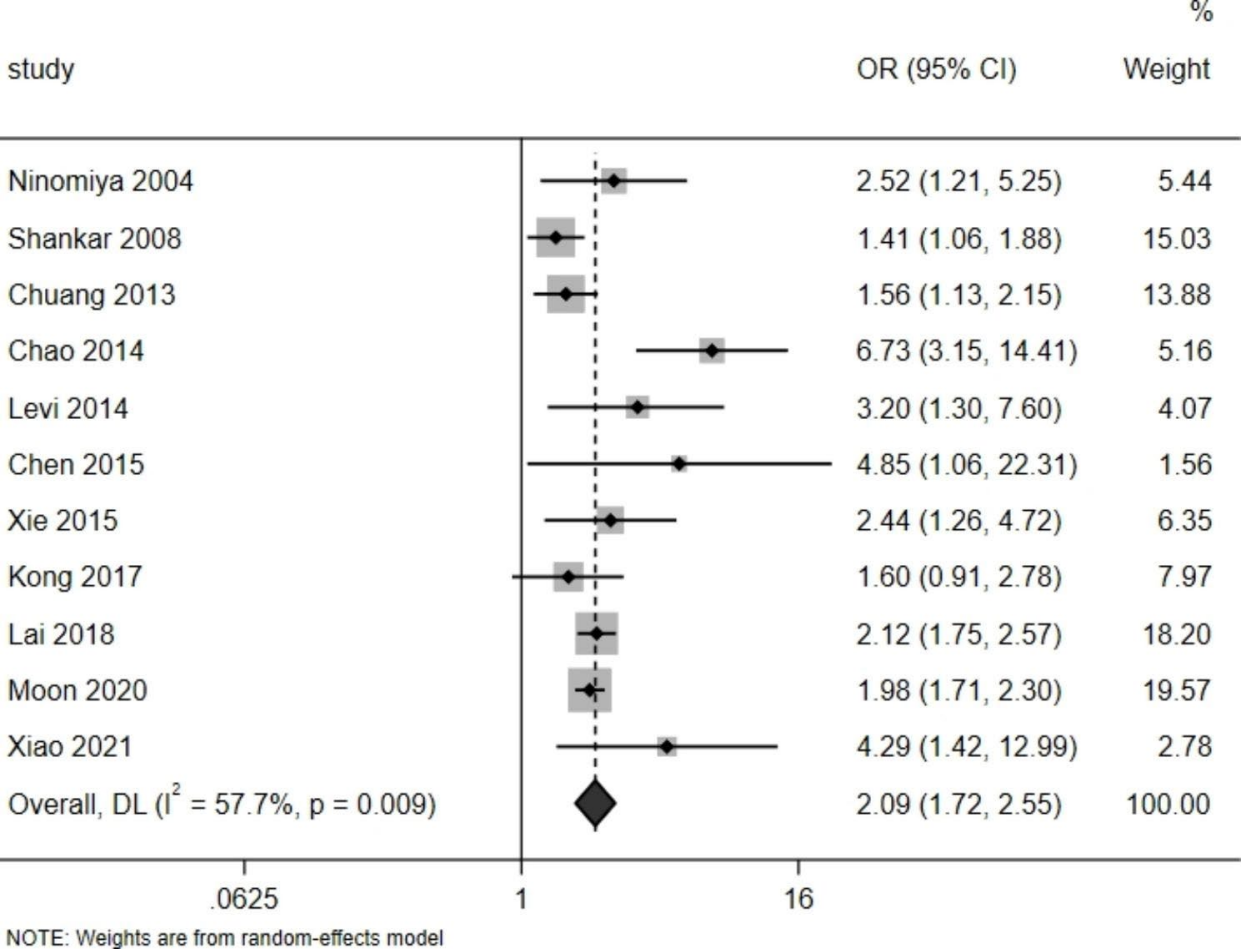
Forest-plot of the meta-analysis that investigated the association of Hcy levels and CKD incidence

**Fig. 3:**
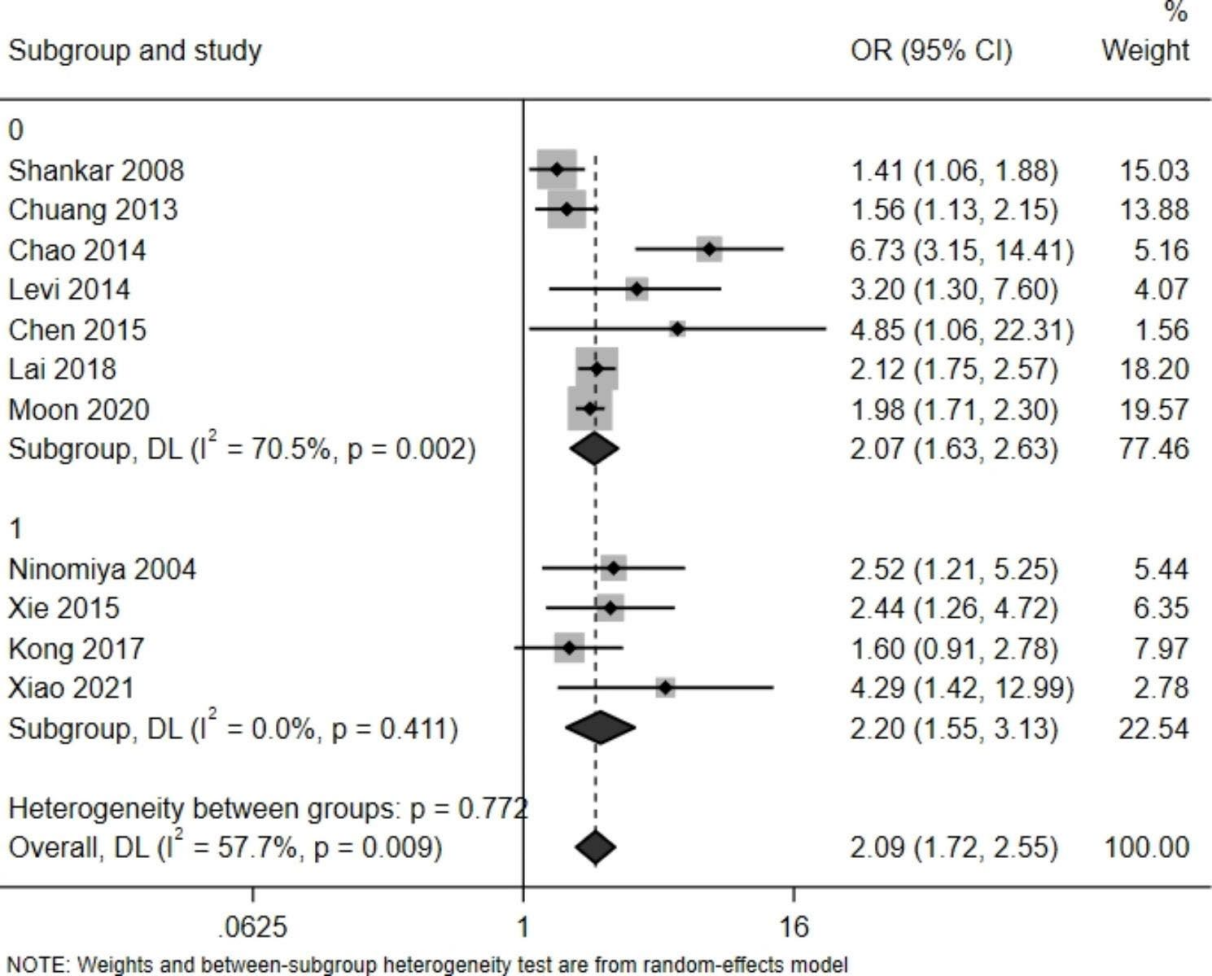
Meta-analysis forest plot for subgroup analysis by study category (0 for a cross-sectional study, 1 for a cohort study)

**Fig. 4:**
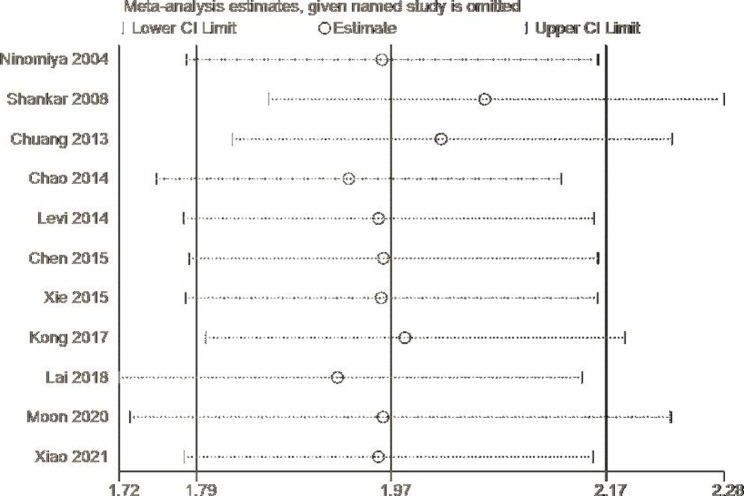
Sensitivity analysis chart

The odds of developing CKD were calculated for each study stratified by different Hcy levels. When a study divided Hcy levels into three groups or more, the OR ratio of the highest level group to the lowest level group was used as the OR of the study. The results of our pooled analysis using a random-effects model showed that there was a significant positive association between Hcy and the incidence of CKD (pooled OR, 2.09; 95% CI, 1.72–2.55), with a significant heterogeneity observed between studies (P < 0.01, I^2 = 57.7%) (Fig. 2).

**Fig. 5:**
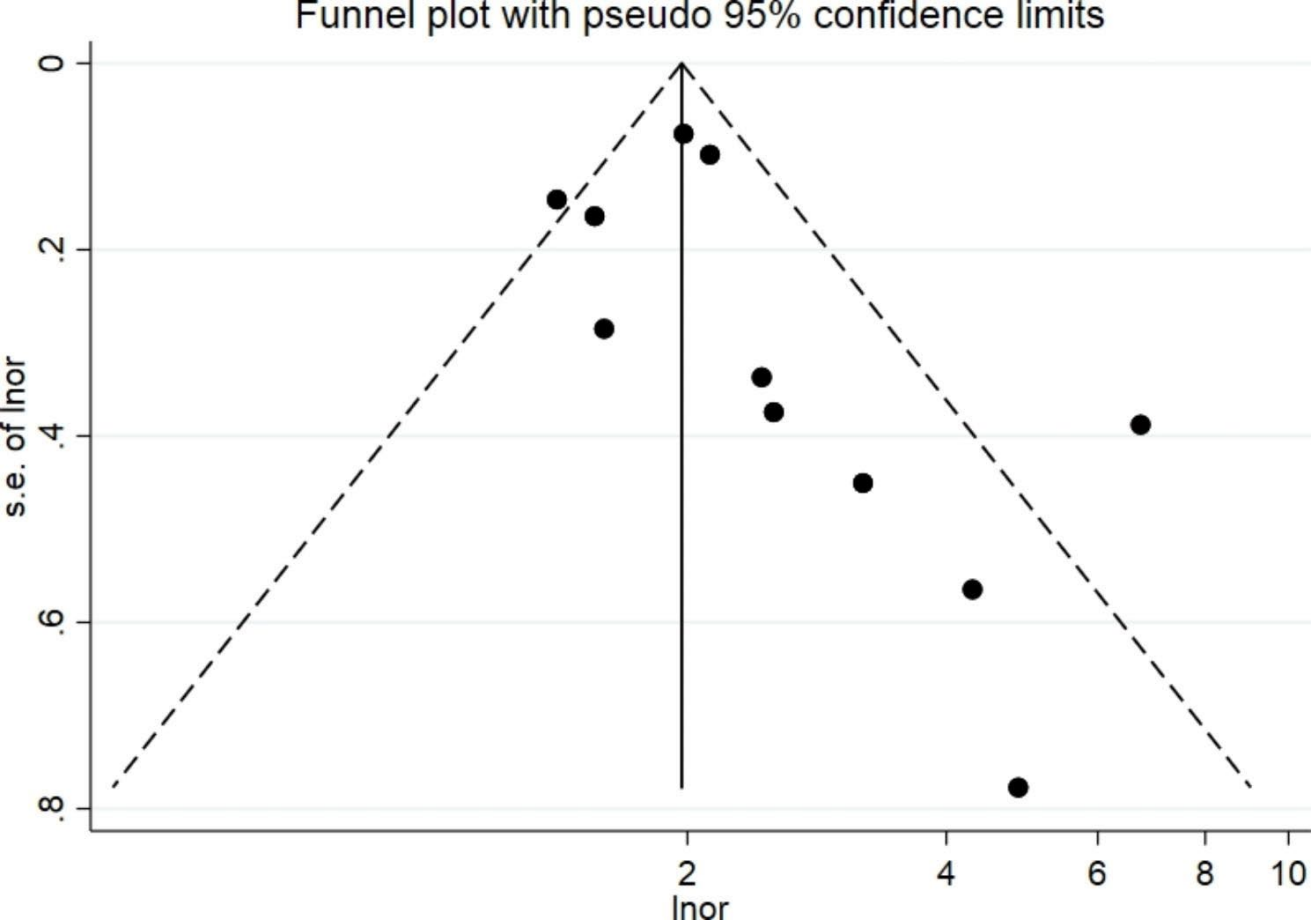
Funnel plot of the association between Hcy and new-onset CKD.

**Fig. 6:**
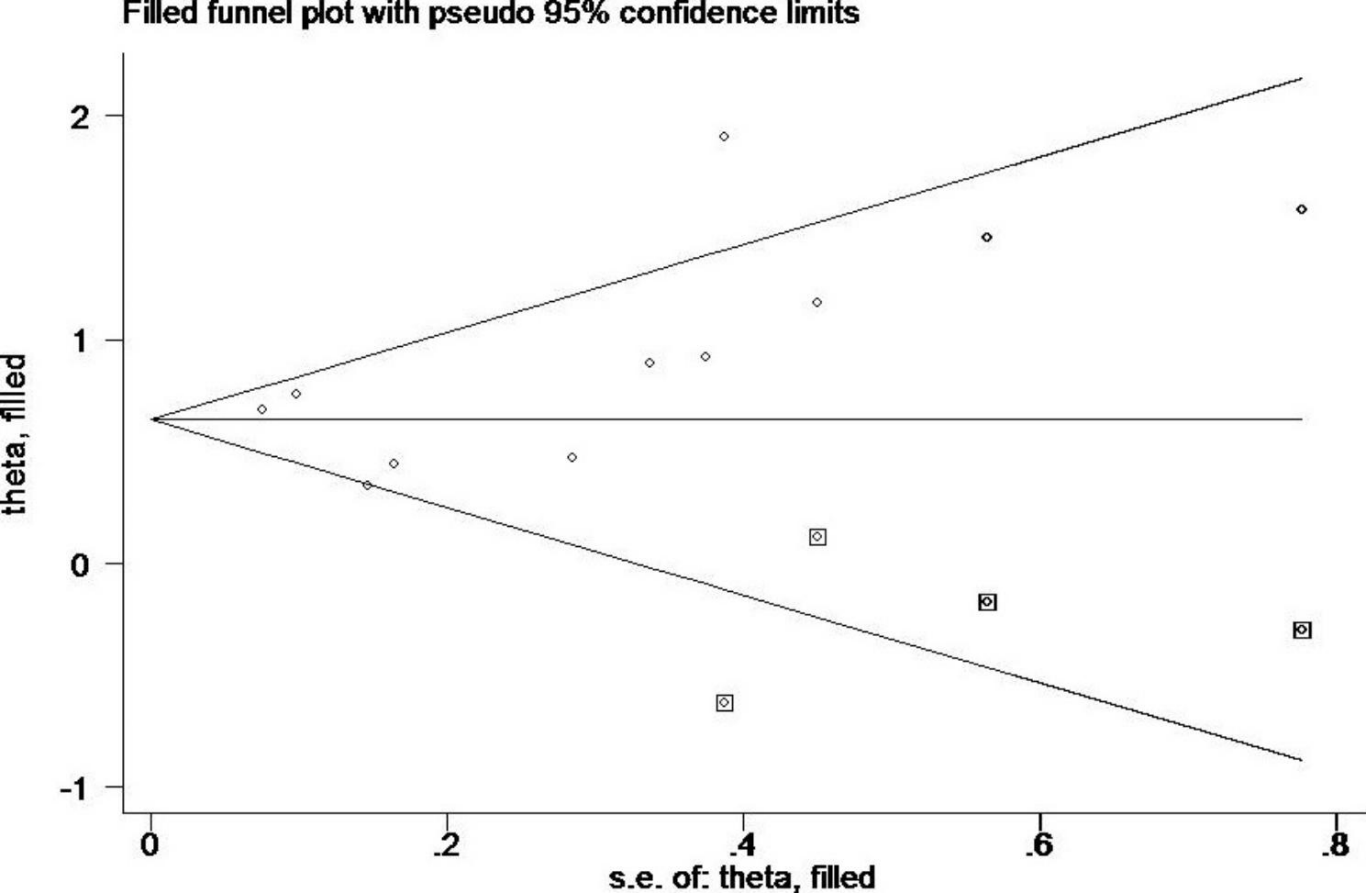
Trimming and filling chart

To search for the sources of heterogeneity we also performed subgroup analyses of cross-sectional studies and prospective cohort studies (Fig. 3). A cross-sectional study was found to be a source of heterogeneity (P < 0.01, I^2 = 70.5%). We also performed a meta-regression analysis, with the results indicating that heterogeneity was caused by the design of the study (P = 0.83 i.e., > 0.05). To further examine the impact of a single study on the total effect we conducted a sensitivity analysis, as shown in Fig. 4.

### Publication bias

The funnel plot analysis qualitatively revealed an asymmetric shape (Fig. 5), suggesting that the association between Hcy levels and CKD incidence may be subject to publication bias. However, the Egger test showed no publication bias (P = 0.155., the Begg test showed publication bias (P = 0.020). To determine whether the combined effect size was stable, an effect size analysis was performed by trimming and filling. The results were stable (P = 0.00) as the random effect model and fixed model results show stable effects (Fig. 6).

## Discussion

We combined the results of 11 studies and found that people with hyperhomocysteinemia were more likely to develop CKD than those with normal Hcy levels (pooled OR, 2.09; 95% CI, 1.72–2.55). Some studies [25] have shown that Hcy is a risk factor that predicts decreased kidney function. People with a Hcy level > 15 µM were more likely to be associated with an eGFR < 60 mL/min or have proteinuria. The following studies also support our conclusions. Kai et al. found that hyperhomocysteinemia-induced podocyte apoptosis played an important role in renal injury in Cbs+/- mice [26]. Jan et al. also considered [27] that hyperhomocysteinemia was a recognized effective independent risk factor for degenerative diseases including CKD. In addition, Hcy has been shown to induce endothelial dysfunction by inhibiting endothelial cell (EC) proliferation and promoting an inflammatory response. At the same time [28, 29] under conditions of hyperhomocysteinemia, Hcy load leads to the expression of endoplasmic reticulum stress genes, resulting in cell damage in cultured podocytes. This suggests that there is a relationship between Hcy and kidney injury, which eventually leads to focal symptomatic or global glomerulosclerosis, tubular atrophy, interstitial fibrosis, and a decreased GFR. Kon et al. revealed in a prospective cohort study that hyperhomocysteinemia increases the risk of decreased eGFR [17]. Ye, Z., et al. used multivariate linear regression analysis to show tha that plasma homocysteine was only associated with eGFR, and serum homocysteine levels were associated with impaired renal function in patients with CKD [9].

CKD is a major global public health problem, with its incidence expected to continue to rise as the incidence of diabetes and hypertension (the main causes of CKD) continue to rise globally [2], The increasing incidence of CKD will certainly lead to a continued increase in the global burden of CKD. A study [30] reported a high global prevalence of CKD, with an estimated global prevalence ranging from 11 to 13%, most of which was stage 3 CKD. The estimated global number of patients with end-stage renal disease (ESKD) requiring renal replacement therapy is estimated at 4.902–7.083 million. CKD also directly affects the global burden of morbidity and mortality. The large number of deaths due to a lack of renal replacement therapy in developing countries, and the large increase in ESKD patients in the future, will place a huge economic burden on even the wealthiest countries [31]. CKD is a silent epidemic because the disease can progress quietly to an advanced stage, and early detection is critical for timely intervention [32]. Therefore, it is very important to find an indicator that can predict the incidence of CKD in the early stages.

At present, Evaluating glomerular filtration rate(GFR) based on Cystatin C has more advantageous than Creatinine [33]. Cystatin C is closely related to homocysteine in patients with CKD. In the absence of stage 3 or 4 CKD, The incidence of elevated homocysteine level in patients with elevated serum Cystatin C was higher than that in patients with normal serum Cystatin C [34]. Chen, T, et al. found in their research [35] that in patients with CKD, the levels of Cys C and Hcy gradually increase as renal function deteriorates. Additionally, the level of creatinine increases with the elevation of Cys C and Hcy levels. Meanwhile, eGFR decreases with the increase of Cys C and Hcy levels. Furthermore, the elevation of Cys C and Hcy is also an independent risk factor for Arteriosclerosis in patients with CKD. Therefore, we hypothesize that Hcy may serve as a good indicator for evaluating the degree of renal function damage in clinical settings. Hcy [36] is a non-protein amino acid and HHcy can affects transcriptional control through changes in histone modification. In addition, HHcy also affects gene expression by changing DNA methylation. Smith et al. [37] reviewed the literature and found that more than 100 diseases or conditions were associated with elevated plasma total Hcy concentrations. A cross-sectional study [3] by Cohen et al. observed that the relationship between Hcy concentrations and CKD was confirmed at all levels of kidney disease, even after adjustment for confounding factors that may affect CKD. The association existed in both men and women.

Due to the significant heterogeneity of this study (I^2 = 57.7%, P < 0.01), we carried out a sensitivity analysis and subgroup analysis to further explore the relationship between Hcy and CKD. The sensitivity analysis showed that the effect of excluding single studies had no significant effect on this relationship. Cross-sectional studies (I^2 = 70.5%, P < 0.01) were found to be the main source of heterogeneity in the subgroup analysis. In addition, the results of cohort studies (I^2 = 0.0%, P = 0.41) suggested that hyperhomocysteinemia may be an independent risk factor for CKD in the general population (pooled OR, 2.2; 95% CI, 1.55–3.13). In a prospective cohort study of 1426 community populations [6] elevated plasma Hcy was found to be an independent predictor of renal function decline and CKD events. Hcy may therefore be a clinically useful tool for predicting the occurrence of kidney disease in the general population. There is substantial evidence [38] that Hcy and/or one of its precursors/metabolites are toxic. Majumder et al. [39] showed that CKD is closely related to cell damage and accumulation of extracellular matrix (ECM) proteins in the glomerular interstitium. Hcy inactivates Akt and activates FOXO1 by dephosphorylating signaling molecules and inducing nuclear translocation of FOXO1, followed by activation of the FOXO1 transcription factor. This leads to induction of apoptosis and synthesis of excess ECM proteins. Parket et al. [40] also considered that eGFR may decrease due to high blood Hcy levels. Trials In the future that consistently reduce Hcy levels may contribute to primary prevention of renal function damage. Therefore, we consider that our study provides credible results that people with a high Hcy level have a higher incidence of CKD.

To our knowledge, only a small number of prospective studies on the general population have investigated whether reducing Hcy levels can prevent the decline in renal function or prevent the risk of developing CKD. Further longitudinal studies on this association that include people with high Hcy levels in the general population may help to elucidate causality and determine whether any interventions such as vitamin supplementation or dietary changes and increased exercise have the potential to reduce Hcy amino acid levels and prevent the occurrence of CKD.

### Strengths and limitations

The role of Hcy in the occurrence and evolution of CKD has initiated many studies in recent years, although the results remain inconclusive due to the complex and bidirectional interaction between changes in Hcy levels and renal function. To the best of our knowledge, this study is the first to systematically evaluate the indicated effect of Hcy levels on the incidence of CKD in the general population.

However, the current study may have limitations. Firstly, most of the included studies were from cross-sectional studies, and we were unable to determine a causal relationship between Hcy and CKD. Secondly, sex comparisons were not performed. Gender differences [41, 42] are known to be critical in many diseases, including CKD, with men more likely than women to develop CKD, possibly due to the direct effect of sex hormones. Various cellular processes are influenced by sex hormones, which regulate the synthesis of various cytokines, vasoactive agents, and growth factors. These effects can alter renal hemodynamics, thereby affecting the progression of renal disease. Thirdly, because different studies have different classification criteria for high Hcy levels this may increase the heterogeneity of the studies. However, due to the small number of studies included in our meta-analysis it was not possible to conduct subgroup analysis for further discussion. Finally, most of the subjects in this study were Asians and accordingly there may be certain limitations in the applicability of our findings to other ethnicities. All of our results would therefore be more convincing if there were more high-quality, large-sample RCTs in this meta-analysis.

## Conclusion

This meta-analysis suggests that people with hyperhomocysteinemia have a higher incidence of CKD. We consider that there may be a causal relationship between high Hcy levels and the prevalence of CKD in the general population. Early detection of population Hcy levels may be helpful for detection of CKD patients.

### Electronic supplementary material

Below is the link to the electronic supplementary material.

Supplementary Material 1

## Data Availability

The datasets used and/or analysed during the current study available from the corresponding author on reasonable request.

## Abbreviations

CKD: Chronic kidney disease
Hcy: Homocysteine
HHcy: Hyperhomocysteinemia
Cystatin C: Cys C
